# Case Report: Split liver transplantation for graft liver failure due to antibody-mediated rejection after immune checkpoint inhibitor therapy

**DOI:** 10.3389/fimmu.2025.1556851

**Published:** 2025-09-01

**Authors:** Hui Tang, Binsheng Fu, Qing Yang, Jia Yao, Kaining Zeng, Xiao Feng, Yang Yang, Shuhong Yi

**Affiliations:** Department of Hepatic Surgery and Liver Transplantation Center, The Third Affiliated Hospital of Sun Yat−Sen University, Guangzhou, Guangdong, China

**Keywords:** hepatocellular carcinoma (HCC), immune checkpoint inhibitors (ICIs), acute graft failure (AGF), antibody-mediated rejection (AMR), split liver transplantation (SLT)

## Abstract

**Objective:**

To explore the clinical experience of split liver transplantation (SLT) as a salvage treatment for acute graft failure (AGF) caused by immune checkpoint inhibitors (ICIs).

**Methods:**

The clinical data of one hepatocellular carcinoma (HCC) patient who underwent two liver transplants were retrospectively reviewed.

**Results:**

The patient received multiple PD-1/PD-L1 inhibitor treatments, with the last one administered 16 days prior to the first transplant. On postoperative day 7, there was a rapid increase in transaminases, indicating acute rejection, which was treated with additional Rabbit anti-human thymocyte immunoglobulin(ATG). On day 14, the patient presented with fatigue, shortness of breath, and abdominal distension. An ultrasound revealed reversed portal vein flow and significant liver enlargement. Given the patient’s deteriorating condition, a rescue second liver transplant (complete right lobe split liver transplantation with middle hepatic vein bipartition/reconstruction) was performed on day 16. The anti-rejection regimen included ATG, Baliximab, Rituximab, glucocorticoids, and intravenous immunoglobulin (IVIG). Postoperative pathology indicated acute liver failure due to humoral rejection. The patient has since been followed for over 12 months, with stable liver function and no signs of rejection or tumor recurrence.

**Conclusions:**

This case highlights the need for cautious use of ICIs before liver transplantation and supports SLT as an effective option in cases of AGF.

## Introduction

The application of immune checkpoint inhibitors (ICIs) in liver transplantation (LT) candidates remains clinically controversial (1, 2), with limited reported cases due to substantiated concerns about graft rejection mechanisms. We present a hepatocellular carcinoma(HCC) patient who developed severe antibody-mediated rejection(AMR) with concomitant hepatic parenchymal necrosis following PD-1/PD-L1 inhibitor therapy prior to initial LT, ultimately requiring emergency salvage transplantation. Given donor organ shortages, split liver transplantation(SLT) was strategically employed a novel approach not previously described in ICI-related graft failure scenarios. This case highlights two critical considerations: (1) the enduring immune activation from ICIs may precipitate both cellular and humoral rejection pathways, and (2) the imperative for protocol optimization regarding ICI-to-transplant intervals. Multicenter studies are urgently needed to establish risk-stratified guidelines for ICI utilization in transplant candidates, particularly regarding optimal washout periods and rejection surveillance strategies.

## Case presentation

A 44-year-old AB/Rh+ male with recurrent hepatocellular carcinoma (HCC) underwent twice LT procedures between November and December 2023. Initial imaging revealed three LI-RADS 5 lesions (all <3 cm) in the right hepatic lobe, staged as T2N0M0 by UNOS-OPTN criteria. Ethical approval was obtained from the Institutional Review Board, with informed consent requirement waived for retrospective analysis. Written consent was specifically obtained for case publication. Donor organs were procured through ethical channels in compliance with Istanbul Declaration guidelines.

### Clinical timeline

2021-11:Laparoscopic left hepatectomy revealed moderately differentiated HCC (5.5 cm, T3N0M0) with left hepatic vein invasion. 2022-07: First recurrence treated with radiofrequency ablation (RFA) and lenvatinib (Lenvima^®^,8 mg once daily). 2023-07: Second recurrence managed with RFA and systemic therapy: regorafenib (Stivarga^®^,160 mg daily) + adebrelimab (PD-L1 inhibitor, 1200 mg every 3 weeks ×3 cycles). 2023-10: Disease progression prompted transarterial chemoembolization(TACE) with mFOLFOX4 hepatic arterial infusion, followed by camrelizumab (PD-1 inhibitor, 200 mg single dose) on 2023-11-06.

### Pre-LT evaluation

All 3 recurrent lesions were located in the right lobe, all less than 3 cm, meeting the Milan criteria. The patient received a total of 3 cycles of PD-L1 and 1 cycle of PD-1 prior to transplantation, with no adverse reactions occurring during administration and no concurrent use of other biologic agents during this period.

The patient underwent a piggyback LT on November 22, 2023 (16 days after the last use of PD-1, Child-Pugh score A, MELD score 6, tumor stage: T3N0M0). The graft was procured from a ABO-compatible DBD donor with cold ischemia time of 312 minutes, no histopathological evidence of steatosis, and without perioperative red blood cell transfusion requirements. The immunosuppressive induction regimen was as follows: intraoperative administration of ATG(Thymoglobuline^®^) 50mg and glucocorticoids(methylprednisolone) 500mg, followed by ATG 50mg daily for two consecutive postoperative days. Methylprednisolone was tapered from 60mg Q6h to 20mg Q6h before discontinuation. Tacrolimus(Prograf^®^) was initiated on postoperative day 2 (2mg Bid daily), with dose adjustments based on trough levels to maintain a target concentration of 7–10 ng/mL. On the first day after surgery, aspartate aminotransferase(AST) was 632 U/L and alanine aminotransferase(ALT) was 265 U/L.The postoperative pathology suggested changes consistent with moderately differentiated HCC.

**Figure 1:**
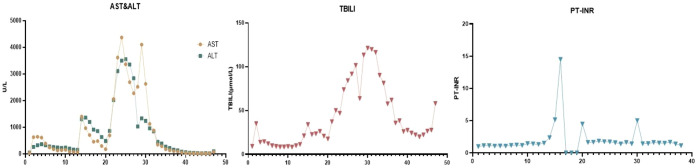
Liver function during treatment. (AST, aspartate aminotransferase; ALT, alanine aminotransferase; TBIL, total bilirubin; PT, prothrombin time).

On postoperative day 7, the ALT increased rapidly to 1300 U/L, indicating acute rejection, for which additional ATG was administered, leading to a gradual decrease in ALT levels. On day 14, an ultrasound showed reversed portal vein flow, significant liver swelling, and poorly perfused hepatic parenchyma, AST up to 3365U/L and ALT 3555U/L, leading to acute fulminant graft failure and transfer to the ICU for plasma exchange and intubated (**Figure 1**). We did not perform a liver biopsy at this stage. On day 14,multiple donor specific antibodies(DSA) against human leukocyte antigen(HLA) class II antigens were detected,indicated AMR.

**Figure 2:**
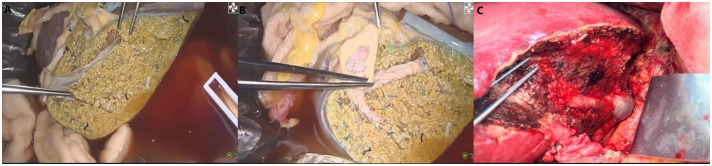
Complete right lobe split liver transplantation with middle hepatic vein bipartition/reconstruction (**A**. MHV is completely bipartited. **B**. MHV reconstructed by arterial patches obtained by deceased donor iliac homograft. **C**. MHV venous drainage after LT).

**Figure 3:**
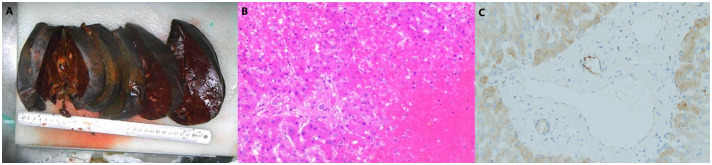
Pathology of salvage split liver transplantation.(**A**.Gross specimen of the liver. **B**.Pathology slide: The extensive degeneration and necrosis of hepatocytes are observed, with some areas retaining only the liver sinusoidal framework. The lesion predominantly involves regions around the central vein, where thrombosis is visible within the central vein, accompanied by surrounding hepatic sinusoidal congestion and iron-containing bile pigment deposition. Hepatocytes in the portal areas are still preserved. Red blood cells, fibrin, and platelet-like substances are also present in the liver sinuses. The portal areas are slightly expanded, with a minimal infiltration of lymphocytes, plasma cells, and neutrophils, but interface inflammation is not observed. There is no significant proliferation or destruction of small bile ducts, and no cholangitis or endothelialitis in the veins is noted. Based on the results of special staining, these findings are considered changes associated with acute liver failure secondary to humoral rejection. **C**. C4d staining: Some large portal vein areas show positive staining, while small portal veins are mostly negative. Overall, there is a significant amount of non-specific staining in hepatocytes.).

On day 16 post-LT, following a thorough evaluation, the patient underwent salvage SLT (complete right lobe split liver transplantation with middle hepatic vein bipartition/reconstruction, **Figure 2**). The second graft was also procured from a O/RH+ blood type DBD donor with cold ischemia time of 453 minutes, no histopathological evidence of steatosis, and perioperative 10U red blood cell transfusion. Given the DSA findings, he was concurrently treated for antibody-mediated and acute cellular rejection. The immunosuppressive induction regimen was as follows: Intraoperatively, ATG 50mg, Baliximab (Simulect^®^) 20mg, and methylprednisolone 500mg were administered. Postoperatively, ATG 50mg was continued for 4 consecutive days, with Baliximab 20mg and Rituximab (Mabthera^®^) 200mg administered on the 4th day. Methylprednisolone was also tapered from 60mg Q6h to 20mg Q6h before discontinuation. Tacrolimus was initiated on second postoperative day(2mg Bid daily), with dose adjustments based on trough levels to maintain a target concentration of 7–10 ng/mL. IVIG 20g Qd from the first postoperation day. On the first day after surgery, AST was 2278U/L and ALT was 2840U/L.Pathology confirmed acute graft failure (AGF) due to humoral rejection, the C4d result (+++). (**Figure 3**).

The patient recovered without complications, was discharged in the second postoperative week, and has since maintained stable liver function with no indications of rejection or tumor recurrence. The early anti-rejection regimen after discharge included Tacrolimus (Prograf^®^), Mycophenolate Sodium Enteric-coated Tablets (Myfortic^®^), and methylprednisolone, which was later changed to Tacrolimus and Sirolimus (Rapamune^®^) after one month. The patient has since been followed for over 12 months, with stable liver function and no signs of rejection or tumor recurrence.

## Discussion

Combination systemic therapies involving ICIs have become increasingly important in the treatment of intermediate and advanced HCC. De Stefano et al. (3) and Woo et al. (4) systematically reviewed case reports and studies published between 2019 and 2024 concerning the administration of ICIs prior to LT. Their analyses revealed substantial variability in outcomes, largely attributable to differences in individual immune microenvironments, pharmacokinetic profiles (e.g., varying half-lives of ICIs), treatment regimens, and interval from therapy discontinuation to transplantation. To date, however, no standardized guidelines or consensus have been established regarding optimal ICIs management in the pre-transplant setting (5).

Although successful LT has been reported following ICIs therapy, acute rejection and graft loss remain major concerns. The incidence of acute rejection among LT recipients ranges from 20% to 50% (6), with graft loss rates of 5% to 10% (7), suggesting higher rejection risks in ICI-treated patients. In our case, the patient developed severe acute rejection, extensive hepatic necrosis, and graft loss after receiving three cycles of adebrelimab and one cycle of camrelizumab shortly before undergoing the first LT. Nevertheless, salvage transplantation was successfully performed via SLT.Many experts have proposed that the risk of post-transplant rejection may depend on the dosage of ICIs and the interval between their discontinuation and LT (3, 4, 8). Insufficient cessation time can lead to insufficient clearance of the final ICI dose, allowing the newly transplanted liver to upregulate PD-L1 and potentially undergo “immune escape,” thereby triggering rejection (2, 9). Although drug intervals typically derive from pharmacokinetic half-lives, high target-binding capacity can persist long after the last dose (**Table 1**). For instance, Tabrizian et al. (10) reported two successful LT cases where nivolumab was discontinued only 1 and 2 days prior to surgery, whereas Nordness et al. (11) described a fatal rejection episode after an 8-day washout. These findings, however, stem solely from case studies and thus lack robust evidence from larger trials. Conversely, Schwacha-Eipper et al. (12) documented a successful transplantation after a 6-week cessation period without notable rejection. In alignment with this, a recent multicenter cohort study by Guo et al. (13) identified a washout of ≥30 days as a significant protective factor. Still, another international retrospective study found that shorter intervals (<30 days and 30–50 days) correlate with elevated rejection rates relative to those exceeding 50 days (14). Therefore, the optimal timing for halting ICI therapy remains controversial, with no established consensus. A widely mentioned guideline suggests waiting at least three half-lives to reduce complications (15).

**Table 1:** PD-1/PD-L1 inhibitor and their half-lives.

PD-1/PD-L1 inhibitors	Trade name	Mechanism	Half-life(days)
Nivolumab	Opdivo	PD-1 Inhibitor	26.7 (FDA 2014)
Pembrolizumab	Keytruda	PD-1 Inhibitor	23 (FDA 2016)
Camrelizumab	Airuika	PD-1 Inhibitor	5.5 (NMPA 2019)
Toripalimab	Tuoyi	PD-1 Inhibitor	12.6 (NMPA 2018)
Sintilimab	Daboshu	PD-1 Inhibitor	19.6 (NMPA 2018)
Tislelizumab	Baizean	PD-1 Inhibitor	13.3 (NMPA 2019)
Penpulimab	Annike	PD-1 Inhibitor	23.3 (NMPA 2021)
Cemiplimab	Libtayo	PD-1 Inhibitor	19.0(FDA 2021)
Avelumab	Bavencio	PD-L1 Inhibitor	6.1 (FDA 2017)
Atezolizumab	Tecentriq	PD-L1 Inhibitor	27 (FDA 2018)
Adebrelimab	Airuili	PD-L1 Inhibitor	13 (NMPA 2023)
Durvalumab	Imfinzi	PD-L1 Inhibitor	18 (FDA 2018)
Ipilimumab	Yervoy	CTLA-4 Inhibitor	15.4 (FDA 2015)

In our case, the patient discontinued camrelizumab a mere 16 days before LT, resulting in severe acute rejection, extensive hepatic necrosis, and avascular thrombosis. These complications underscore the potential risks associated with brief washout intervals. We recommend avoiding LT within one month of stopping ICIs if the patient’s clinical condition permits, and emphasize that the safest window may be as long as three months. On the other hand, some experts caution that premature cessation of ICIs in HCC patients could accelerate tumor progression and diminish access to potentially life-extending transplant benefits.

Acute rejection induced by ICIs can arise not only from T-cell–mediated pathways, but also from AMR, thereby influencing perioperative anti-rejection strategies. In a comprehensive review of ICI-related rejection events in solid-organ transplant recipients, Nguyen et al. (16) reported that although most cases were driven primarily by T-cell responses, 21.4% also showed evidence of concurrent AMR. Yalda et al. (17) documented a patient who developed liver failure and required a second transplant following preoperative ICI therapy; DSA testing revealed elevated DSA levels, prompting plasma exchange and IVIG administration to mitigate the risk of rejection prior to re-transplantation. Mechanistically, the PD-1/PD-L1 signaling axis may confer protection against AMR by controlling T-cell overactivity, ultimately limiting graft injury; however, blocking this pathway can exacerbate immune-mediated damage—especially under conditions of active AMR.

In the case we reported, postoperative pathology indicated an antibody-mediated humoral immune response, suggesting that anti-B-lymphocyte antibodies may be necessary for both prophylaxis and treatment in such patients. To prevent acute rejection following the initial transplantation, during the second procedure we extended the ATG course from 3 to 5 days and added basiliximab and rituximab to concurrently control immune responses mediated by both T cells and B cells. We recommend the management strategies as follows: ①Discontinuation of ICIs: Immediate cessation of ICI therapy is critical to reduce ongoing immune activation. ②Treatment of AMR: A)Plasmapheresis: removes circulating DSAs and immune complexes. B)IVIG: neutralizes DSAs and modulates immune responses. C)Rituximab: a monoclonal antibody targeting CD20 on B cells, used to deplete B cells and reduce antibody production. D)Complement Inhibition: agents like eculizumab (a C5 inhibitor) may be used to block complement-mediated graft injury. E)High-Dose Steroids: to suppress inflammation and immune activation. ③Re-Transplantation: in cases of irreversible graft failure, liver re-transplantation may be the only option. In this case, pre-transplant antibody testing was not performed prior to the initial LT, precluding confirmation of preformed DSA. However, during secondary LT preparation, donor screening using a 1:10 dilution titer effectively excluded DSA-positive candidates. The marked reduction in DSA levels pre-retransplantation suggests this intervention created favorable immunological conditions contributing to the favorable outcome. The implemented DSA-modulation strategy not only informs future studies evaluating the safety of pre-transplant ICI therapy but also provides a framework for investigating rejection patterns specific to transplant recipients with prior immune checkpoint inhibitor exposure (17). However, the risk of recurrent rejection remains high, particularly if the effects of ICIs persist. The prognosis depends on the severity of rejection, the timeliness of intervention, and the availability of a second graft in cases of graft failure. Outcomes are generally poor in cases of severe AMR refractory to treatment, highlighting the need for careful patient selection and monitoring when considering ICIs in transplant recipients.

Furthermore, during this patient’s second transplantation, we utilized SLT due to a shortage of donor livers, completely splitting the donor liver into right and left halves with middle hepatic vein bipartition/reconstruction. This approach allowed us to effectively use one liver to save two patients simultaneously. The patient successfully underwent salvage LT using the right half of the graft and made a good recovery post-operation, demonstrating that SLT can be safely employed in critical cases like this. Currently, there are no reports about SLT being used for rescue LT in acute rejection or graft failure caused by ICIs. This report presents the first documented case, in which we attempted this high-risk procedure amid donor shortages. However, SLT remains a technically complex surgery with significantly prolonged cold ischemia time compared to whole liver transplantation, making it uncommon for urgent retransplantation. In this case, we performed *in situ* splitting to minimize cold ischemia time and adopted midline splitting of the hepatic middle vein to ensure adequate venous drainage of the right lobe. Whether SLT should be recommended for such critical scenarios remains uncertain, and its broader applicability in these contexts requires further investigation with more case studies.In conclusion, graft liver failure due to humoral rejection after ICI therapy is a serious and challenging complication. It underscores the need for careful consideration of the risks and benefits of ICIs in transplant recipients, as well as the importance of early recognition and aggressive management of rejection episodes. Multidisciplinary collaboration and further research are essential to improve outcomes in this complex clinical scenario.

## Data Availability

The raw data supporting the conclusions of this article will be made available by the authors, without undue reservation.
